# Adequacy, Satisfaction, and Factors Associated With Information Provided During Consent: A Cross-Sectional Study Among Prosthodontic Patients at Selected Dental Facilities in Kampala, Uganda

**DOI:** 10.7759/cureus.77189

**Published:** 2025-01-09

**Authors:** Barbara Ndagire, John Barugahare, Charles M Rwenyonyi, Janet Nakigudde, Sudeshni Naidoo

**Affiliations:** 1 School of Dentistry, Makerere University, Kampala, UGA; 2 Clinical Epidemiology Unit, Makerere University, Kampala, UGA; 3 Department of Philosophy, Makerere University, Kampala, UGA; 4 Department of Psychiatry, Makerere University, Kampala, UGA; 5 Faculty of Dentistry, University of the Western Cape, Cape Town, ZAF

**Keywords:** adequacy, dentists, information, informed consent, satisfaction

## Abstract

Background

In dentistry, the practice of informed consent is crucial, more so for invasive procedures such as fixed prosthodontic treatment. Patients should have adequate information if they are to play a significant role in making decisions that reflect their values and preferences.

Objective

The objective of the present study was to determine the adequacy and factors associated with information provided during the informed consent process for fixed prosthodontic treatment among patients attending selected dental facilities in Kampala, Uganda.

Methods

The present study was a facility-based, cross-sectional study conducted between September 2023 and March 2024 at three dental facilities within tertiary-level hospitals in Kampala, the capital city of Uganda. The participants were patients aged 18 years and above who attended the selected dental facilities, needed fixed prosthodontic treatment, received information regarding the same, and consented to participate. Data were collected using three methods: administering a semi-structured questionnaire to patients, observation of the consent process, and a review of the patient records. Four research assistants administered a questionnaire to the participants in the form of an oral interview. It included items about participants’ socio-demographic characteristics, service-related factors, elements of information provided, and patient satisfaction. Descriptive statistics, Chi-square/Fisher’s exact, and multivariate logistic regression were used to analyze the data. The significance level was set at p <0.05.

Results

The median age of the participants was 30 years (interquartile range (IQR) 24-38 years). Almost all (99.0%) had a formal education with more than two-thirds (70.6%) having a degree or diploma. The majority (>80.0%) needed treatment with a crown. All participants received the information verbally and only 4.2% reported having received written information. Overall, just over half (52.6 %) received adequate consent information, though, most participants were satisfied with the information provided. About a third (38.8%) of them received information regarding the probable risks or postoperative complications (29.7%) of the treatment they were to undertake. There was variation in the components of information provided by the dentists during the informed consent process and there were no treatment-specific consent forms used. In multivariate analysis, patients who had the opportunity to ask questions and those who had received information at a prior appointment before treatment were positively associated with adequate information.

Conclusion

The present study reported on the inadequacy of information provided during the informed consent process for fixed prosthodontic treatment. It is recommended to develop and use a standardized informed consent document that includes all pertinent information, together with additional visual aids to enhance the delivery of information. In addition, it is suggested to give patients the opportunity to ask questions and clarify any information provided.

## Introduction

Informed consent is essential in clinical practice, particularly for ethical, legal, and administrative purposes [1]. It is the systematic process of disclosure of relevant information by the attending clinician that a competent patient will appreciate by understanding the facts enabling him/her to make voluntary and informed decisions [1-3]. As such, patients should have adequate information if they are to play a significant role in making decisions that reflect their values and preferences, while clinicians play a key role as educators in the consent process [4,5].

In dentistry, the practice of informed consent is crucial, more so for invasive procedures such as fixed prosthodontic treatment due to the irreversibility, complexity, high costs, and technicality of the procedures required, high expectations of the patients, and the availability of various treatment options [6,7]. Inadequate information provided to patients during the informed consent process for fixed prosthodontic treatments has been associated with negative outcomes including low patient satisfaction, poor adherence to treatment, patient regret, and litigations [6,7].

There are varying views regarding the information a patient needs to receive during consent; these opinions include the reasonable patient, reasonable physician, and subjective standards [8,9]. However, several ethical guidelines state that the basic components of information to be provided during the consent process should include a description of the problem, procedures to be performed and available alternatives, the anticipated risks, benefits, and probable complications, as well as facilitating the patient to ask questions [4,9]. In Uganda, the provision of adequate information during the informed consent process is a legal requirement as per the Patient’s Charter and Ethical Code of Conduct enforced by the Uganda Medical and Practitioners Council [10,11]. Article 10 of the Patient’s Charter states that “Every patient has the right to be given adequate and accurate information about the nature of one’s illness, diagnostic procedures, the proposed treatment for one to make a decision that affects any one of these elements”.

Available literature on the adequacy of informed consent in studies in dental and other medical specialties highlights serious shortcomings indicating that actual practices are far from ideal [4,12-16]. The evidence suggests that patients have some general information or idea about the procedures they are to undertake, but may not be aware of alternative treatment options, risks, or complications of the procedures [12-15,17]. In addition, available literature [17,18] reveals that most patients are satisfied with the information received during the consent process despite the adequacy of the information. The limited literature from Uganda also reveals deficiencies in the consent process [2,19]. Improvement in the informed consent process may be achieved through the recognition of the gaps that exist and designing appropriate interventions. Therefore, the present study aimed to determine the adequacy, patient satisfaction, and factors associated with information provided during the informed consent process for fixed prosthodontic treatment among patients attending selected tertiary dental facilities in Kampala, Uganda.

## Materials and methods

Study design

This was a facility-based cross-sectional study conducted between September 2023 and March 2024 at three dental facilities within tertiary-level hospitals in Kampala Capital City Authority (KCCA). The present study was part of a cross-sectional survey to determine the adequacy of information provided and assess patients’ understanding of consent information provided before fixed prosthodontic treatment among patients attending selected dental facilities in KCCA, Uganda. The study was approved by the Makerere University School of Medicine Research Ethics Committee (Reference number: Mak-SOMREC-2022-418, dated May 3, 2023).

Study setting

Kampala is the capital city of Uganda and the most populated urban center in Uganda with a resident population of about 1.5 million [20]. Uganda has a mixed healthcare system comprising public, private not-for-profit, and private-for-profit health facilities. Purposive sampling was used to select three tertiary-level hospitals, aiming at dental facilities that have high patient volumes, and offer fixed prosthodontic treatment. The selected hospitals were Mulago National Referral Hospital (MNRH), Mengo Hospital, and Makerere University Dental Hospital. MNRH and Makerere University Dental Hospitals offer public and private dental services while Mengo Hospital is a private not-for-profit facility.

Study population and selection criteria

The study was conducted among patients who attended the selected dental facilities during the period of data collection and had treatment plans requiring fixed prosthodontics. All patients aged 18 years and above who needed fixed prosthodontic treatment, received information regarding the proposed treatment, and were willing to participate were included in the study. Patients undergoing treatment for any mental health condition, those with professional dental training, or were unable to speak or understand English or Luganda were excluded from the study.

Sample size determination and sampling procedure

The sample size was estimated using the formula of $N=\frac{z_{\alpha/2}^{2}P(1-p)}{d^{2}}$ [21]. Assuming 50% as the expected prevalence (P) for adequacy of consent, z value of 1.96 at a 95% confidence interval, and precision of 5%, the sample size was estimated at N=384.

A proportional-to-sample technique was used to determine the number of patients to be selected to ensure that each hospital was represented adequately in the sample by considering the average monthly and then estimating the annual number of patients who receive fixed prosthodontic treatment at the selected hospitals (n0). The number of patient samples (nk) from each hospital was calculated using the formula below: $nk=\frac{no}{N}*n$; Where; nk: number of patients sampled from each hospital, i.e., Mengo, MNRH, and Makerere University Dental Hospital; n0: Estimated annual number of patients from each hospital, N: Total annual number of patients for the three hospital registries. n: Sample size for the study (384). Considering that about 300 patients receive fixed prostheses at Mengo Hospital annually, MNRH treats approximately 379 from the private and public dental facilities, and Makerere University Dental Hospital treats about 485 patients. We proportionally allocated 99 patients to Mengo Hospital, 125 patients to MNRH, and 160 patients to Makerere University Dental Hospital based on the figures. At each hospital consecutive recruitment was used to select participants till the respective sample size was obtained. About five patients were approached for the interview and declined to participate in the study because of time constraints.

Data collection

Data Collection Procedure

Data were collected using three methods that included administering a semi-structured questionnaire to patients, observation of the consent process, and review of the patient records. Four research assistants who were trained dentists obtained consent from participants and collected the data. The research assistants had no involvement in the delivery of dental care to the study participants. Patients who consented to participation were requested to allow an interview to administer the questionnaire either immediately or within 1-2 days of receiving information regarding their treatment. The participants were interviewed in a private area to ensure confidentiality.

Semi-structured Questionnaire

A questionnaire was administered to the patients in the form of an oral interview in either English or Luganda languages as the research assistant recorded the responses. The questionnaire was adapted from two similar studies [22, 23] with modifications to focus on fixed prosthodontic treatment. The questionnaire for assessing the adequacy of information consisted of two sections: 1) socio-demographic information and service-related factors, 2) elements of consent information, the extent to which patients were satisfied with the information provided, and any further information they might have wished to know. Responses to the items in section 2 were recorded using yes (1), and no (0) options. The questionnaire for patients is attached in the appendices (Table 6).

Quality Control

Prior to the commencement of the study, the four research assistants were trained on the informed consent process in dental care, with a focus on fixed prosthodontic treatment by the principal investigator (BN). Before the main study, the questionnaire was piloted among a convenience sample of 15 patients at Makerere University Dental Hospital (MUK-DH) to gain feedback on the overall acceptability of the questionnaire in terms of language, clarity, and length and determine the reliability of the questionnaire.

Direct Observation of Consent Process and Document Review

At each of the selected dental facilities, a research assistant requested permission from both the patient and dentist to observe the consenting process for five patients. After completion of the patient’s appointment, the research assistant would record the information observed during the consent process and review the patient's record card using a checklist with a yes-or-no option. In order to reduce the Hawthorne effect, the research assistants established a rapport, addressed any misconceptions about the study, and forged relationships with the dentists, before collecting data.

Study variables

Dependent variables were adequacy of consent information and satisfaction with consent information. Independent variables included socio-demographic factors (age, sex, marital status, occupation, level of education attained, and the ability to read and write), and service-related factors (timing of providing information, designation of the person who provided consent information, the language used in consent process and method used to delivery information and history of similar treatment).

Data management and analysis

The data files were securely stored in a locker in the principal investigator’s offices while electronic files were kept on a password-protected laptop accessible only to the study team. Quantitative data was entered into a computer using Epi-data software and analyzed using STATA, version 14. Data were summarized using descriptive statistics. Adequacy of consent information was computed by determining the total number of affirmative responses for information received by the patient. It was categorized into a binary variable: where adequate information was considered if the participant had affirmative responses to 8 or more of the 10 items, and inadequate information if the participant provided affirmative responses to less than eight items. Chi-square/Fischer’s exact tests were used to determine the associations between categorical variables. Bivariate Poisson regression was used and variables with a p-value of ≤ 0.2 were entered into a multivariate model. Multivariate Poisson regression was used to determine the factors associated with the provision of adequate information. The level of significance was set at 5%. Content analysis was used to analyze responses to the open-ended items regarding any other information patients would have liked to know regarding their treatment and data from observations and document review.

## Results

Socio-demographic characteristics of the study participants

The median age of the participants was 30 years with an age range from 18 to 69 years (interquartile range (IQR) 24-38 years). More than half (59.4%) were female. Almost all (99.0%) had a formal education with more than two-thirds (70.6%) having a degree or diploma. More than two-thirds (67.2%) had some form of employment (Table 1).

**Table 1:** The frequency distribution of participants according to their socio-demographic characteristics (n=384) MNRH: Mulago National Referral Hospital; MUK: Makerere University Kampala

Characteristic		Frequency n (%)
Facility	Mengo Hospital	99 (25.8)
	MNRH	126 (32.8)
	MUK Dental Hospital	159 (41.4)
Sex	Female	228 (59.4)
	Male	156 (40.6)
Age category (in years)	18-29	187 (48.7)
	30-35	145 (37.8)
	>36	52 (13.5)
Marital Status	Single	214 (55.7)
	Married	156 (40.6)
	Widowed/Separated	14 (3.7)
Religion	Catholic	127 (33.1)
	Protestant	119 (31.0)
	Muslim	47 (12.2)
	Other	91 (23.7)
Highest level of education	None	4 (1.0)
	Primary	18 (4.7)
	Secondary	91 (23.7)
	Tertiary	271 (70.6)
Ability to read and write	Yes	375 (97.7)
Occupation	Unemployed	18 (4.7)
	Studying	108 (28.1)
	Business/Self-employed	109 (28.4)
	Civil/Public service/Formal employment	119 (31.0)
	Farming or other	30 (7.8)

Service factors related to the consent process

The majority (>80.0%) of the participants needed a crown, and it was their first experience to receive such a treatment procedure. Most (89.1%) participants stated that the information was given to them by the dentists who would perform the treatment. All participants received the information in verbal discussion and less than one-third (26.0%) of them reportedly used visual aids in the discussion. Only 4.2% received written information and just less than a quarter (24.2%) signed a consent form (Table 2). There were significant differences in the responses of the participants from different hospitals concerning the time at which patients received the information and the language used in the discussions.

**Table 2:** The frequency distribution of participants according to service factors related to the informed consent process FPT: fixed prosthodontic treatment; Chi-square/Fischer’s exact tests were used. *Fischer’s exact used, bold signifies statistically significant, p<0.05

Service-Related Factor		Dental Hospital				
		Overall Frequency	Mulago NRH	Mengo DH	MUK-DH	Chi
		n (%)	n (%)	n (%)	n (%)	
Type of FPT	Crown	342 (89.1)	108 (85.7)	90 (90.9)	144 (90.6)	0.466*
	Bridge	29 (7.5)	14 (11.1)	5 (5.0)	10 (6.3)	
	Other	13 (3.4)	4 (3.2)	4 (4.0)	5 (3.1)	
Cadre who provided consent information	Dentist who will perform the treatment	330 (85.9)	114 (90.4)	81 (81.8)	135 (84.9)	0.055*
	Other dentists in the practice	39 (10.2)	6 (4.8)	16 (16.2)	17 (10.7)	
	Chair side assistant or dental nurse or other	15 (3.9)	6 (4.8)	2 (2.0)	7 (4.4)	
Time of consent/Timing of providing consent information	Prior appointment	225 (58.6)	74 (58.8)	80 (80.8)	71 (44.7)	<0.001*
	Before starting the procedure on the day of treatment	111 (28.9)	33 (26.2)	8 (8.1)	70 (44.0)	
	While conducting treatment	16 (4.2)	7 (5.6)	2 (2.0)	7 (4.4)	
	During several occasions/More than one of the occasions above	32 (8.3)	12 (9.4)	9 (9.1)	11 (6.9)	
Language used when providing explanations	English	173 (45.0)	75 (59.5)	43 (43.4)	55 (34.6)	<0.001
	Local language	37 (9.6)	12 (9.5)	12 (12.1)	13 (8.2)	
	Both	174 (45.3)	39 (31.0)	44 (44.4)	91 (57.2)	
How was the information given to you	Verbal discussion only	270 (70.3)	88 (69.8)	62 (62.6)	120 (75.5)	0.089
	Visual or written information also used	114 (29.7)	38 (30.2)	37 (37.4)	39 (24.5)	
Signing consent form	Yes	93 (24.2)	10 (7.9)	13 (13.1)	70 (44.0)	<0.001

Adequacy of consent information and patient satisfaction

Most (> 80.0%) participants reported having received information regarding the nature of the problem, the necessity of having the treatment, details of the planned procedure, who would perform it, potential benefits, and costs, and were allowed to ask questions. About one-third received information regarding the probable risks (38.8%) or postoperative complications (29.7%) of the treatment they were to undertake. Less than one-third of them signed a consent form (Table 3). Overall, about half (52.6 %) of the participants received adequate consent information. Most (81.7%) of the participants were satisfied with the information received (Table 3).

**Table 3:** The frequency distribution of the participants according to consent information provided to the participants based on affirmative responses at the selected dental facilities (n=384)

Characteristic		Number	Percentage
Informed about the diagnosis	Yes	375	97.7
Informed of why it is necessary to have the treatment	Yes	375	97.7
Informed about details of the planned procedure	Yes	310	80.7
Informed who would perform the procedure	Yes	350	91.2
Informed of the potential benefits of the treatment	Yes	349	90.9
Informed of the potential risks and inconveniences of the procedure	Yes	149	38.8
Informed of any postoperative complications	Yes	114	29.7
Informed about any other alternative treatment options	Yes	247	64.3
Informed about the cost of the treatment	Yes	345	89.8
Informed about how long it is going to take to complete the procedure and your treatment	Yes	273	71.1
Given a chance to ask questions about the treatment	Yes	337	87.8
Told that they had the right to refuse or defer the decision to have this treatment	Yes	328	85.4
Given a consent form to sign	Yes	93	24.2
Degree of satisfaction with the information you received before this treatment	Very satisfied	128	33.3
	Satisfied	185	48.2
	Neutral/ No opinion	61	15.88
	Low	9	2.34
	Very low	1	0.26

Other information the patients would have liked to receive

**Figure 1:**
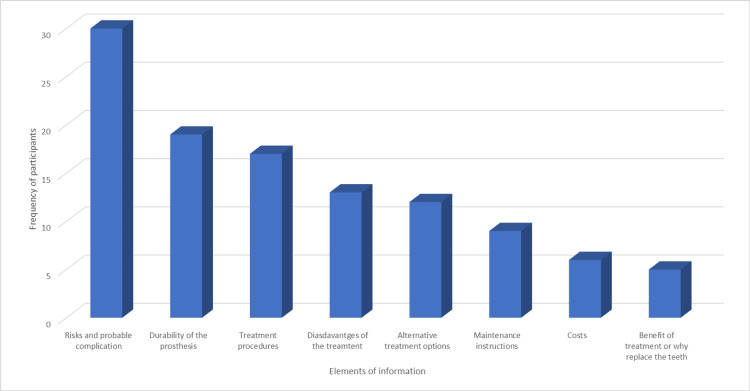
Frequency distribution of elements of information patients would like to know

In response to the open-ended item, the most frequent elements of the other information patients would have wanted to know were risks and probable complications, durability or longevity of the prosthesis, treatment procedures, disadvantages of the treatment, and alternative treatment options (Figure 1).

Relationship between the adequacy of information provided and patient satisfaction

There was a significant association between the adequacy of consent information received and participants’ satisfaction, (p <0.001) (Table 4).

**Table 4:** The frequency distribution of participants according to the relationship between the adequacy of information provided and patient satisfaction Analysis done using chi, p-valve <0.05 considered significant.

	Degree of satisfaction with the information received						
	Very satisfied	Satisfied	Neutral	Low	Very low	No opinion	Total
	n (%)	n (%)	n (%)	n (%)	n (%)	n (%)	
Adequate information provided	95 (47.0)	93 (46.0)	11 (5.5)	3 (1.5)	0 (0.0)	0 (0.0)	202
Not adequate information	33 (18.1)	92 (50.6)	45 (24.7)	6 (3.3)	1 (0.6)	5 (2.8)	182
Total	128 (33.4)	185 (48.3)	55 (14.4)	9 (2.4)	1 (0.3)	5 (1.3)	384

Factors associated with having received adequate information

In multivariate analysis, patients who had the opportunity to ask questions and those who received information at a prior appointment were positively associated with adequate information (Table 5).

**Table 5:** Factors associated with the provision of adequate information PR: prevalence ratio; bolded at bivariate analysis was considered for multivariate analysis; p-value <0.05 was considered significant; Poisson regression was used for analysis.

Factor		Prevalence N=384	Bivariate		Multivariate	
		n (%)=384	PR	p-value	PR	p-value
Facility	Mulago/Naguru hospitals	128	Reference	Reference	Reference	Reference
	Makerere university	159	0.9477	0.671	1.0617	0.630
	Mengo hospital	96	0.8381	0.118	0.8410	0.122
Cadre who provided information	Dentists who will treat	330 (85.9)	Reference	Reference		
	Another dentist	39 (10.2)	0.908	0.577		
	Other cadre in practice	15 (3.9)	0.746	0.360		
Time when information was provided	Prior appointment	224 (58.5)	Reference	Reference	Reference	Reference
	Before starting the procedure on the day of treatment	111 (29.0)	1.269	0.022	1.4821	<0.001
	While conducting treatment	16 (4.2)	1.314	0.185	1.385	0.095
	During several occasions	32 (8.3)	1.183	0.326	1.228	0.213
Language used	English	173 (45.1)	Reference	Reference	Reference	Reference
	Local language	37 (9.6)	0.716	0.111	0.821	0.314
	Both	174 (45.3)	0.903	0.306	0.926	0.435
Had an opportunity to ask questions	No	47 (12.2)	Reference	Reference	Reference	Reference
	Yes	337 (87.8)	3.382	<0.001	3.416	<0.001
Use of visual aids	No	284 (74.0)	Reference	Reference		
	Yes	100 (26.0)	1.063	0.571		

Results for document review and observation of the consent process

Most participants received information from their dentists in an oral discussion. There was variation in the elements of information provided during the consent process. Most of the patients received information regarding the diagnosis, reason for the treatment, description of the procedures, expected time to complete treatment, and costs and they were given an opportunity to ask questions. A few patients received information regarding benefits, foreseeable risks or discomforts, or alternative treatment options.

Information captured on the patient record cards included diagnosis, treatment, and costs. No treatment-specific consent forms were available or signed for patients observed who needed crowns or bridges. Patients signed blanket consent to affirm their acceptance to receive any treatment at two of the dental facilities (Makerere University Dental Hospital and MNRH) on the first visit.

## Discussion

The present study provided baseline information necessary for understanding the current practices regarding information disclosure during the consent process for fixed prosthodontic treatment at selected dental facilities in Kampala Uganda. The findings indicated that only about half the participants received adequate information. However, the majority were satisfied with the information received. The study findings are consistent with several studies reporting that less than 50% of patients received adequate information about their treatment [13,14,23]. Tejaswi et al. observed that the detailed procedure was explained to only 14.5% of the patients though 96.5% of them were satisfied with the information given during informed consent for cesarean section at a tertiary care center in India [13]. Furthermore, Chane et al. found that only 8.1% of the patients received the minimum required components of information which included details about the procedure, risks, benefits, and alternatives to the procedure [14]. On the contrary, a study by Patil et al. found that most (> 90%) of surgical patients at a tertiary care center in India reported having received information regarding most components of consent information [18]. In this study, inadequate disclosure may be linked to the absence of standardized consent forms, as most participants reported receiving verbal information only, without signing treatment-specific consent forms. This lack of information may hinder patients’ ability to make informed treatment decisions. Furthermore, providing adequate information is essential for valid informed consent, and dentists have an ethical obligation to do so [9].

According to the study’s findings, the majority of patients were given information regarding their dental problem, need for treatment, treatment procedures, and its potential benefits. However, only about one-third of the participants reported having been informed about the potential risks or complications of fixed prosthodontic treatment. Several studies [4,14,24] in different specialties have obtained comparable findings. For instance, Chane et al. found that more than 55% of the patients were told their diagnosis, and the benefits of treatment, though only 26.7% of them had received explanations about the risks of the procedure [14]. On the contrary, a study conducted among patients at a tertiary hospital in India observed that a substantial (41.2%) proportion of them reported having received information about possible complications. Variation in the elements of information provided during consent may be due to a lack of standardized consent forms as only 4.2% of the participants received any written information. However, dentists have an obligation to disclose balanced and accurate information including both the benefits and risks of procedures and selective truth-telling must be avoided during the consent process [8]. Therefore, patients should be informed about the material risks of procedures including the most common, serious, and relevant risks [25]. In addition, patients should have adequate information if they are to play a significant role in making decisions that reflect their own values and preferences [4,5].

In the present study, information about alternative treatment options was provided to about two-thirds of the patients. These findings differ from results observed by Tamire and Tesfaw [26] and Patil et al. [18], where only 40.7% and 47.5% of the patients, respectively, received information regarding alternative treatments. Patients, on the other hand, have the right to be informed of all the available information about their treatments including the treatment risks, benefits, and alternative treatment options [16]. Only after receiving detailed information, can the patients freely explore all the possible treatment alternatives and work with the dentist to choose the most appropriate option for them. Therefore, we propose that such information be offered to all patients, to facilitate their active participation in shared decision-making, rather than just agreeing with the dentist's choice or signing a consent form.

More than three-quarters of the participants reported that they were given an opportunity to ask questions and this was positively associated with receiving adequate information. This finding is consistent with some recent studies [15,17], that found that the majority (above 80.0%) of patients were given the chance to ask questions. Conversely, a study in Ethiopia by Chane [14] reported that about half (51.1%) of the patients had an opportunity to ask questions. However, the principles of medical ethics emphasize that no treatment should ever be undertaken without giving a patient the opportunity to ask questions and/or raise any concerns or fears [9]. Allowing patients to ask questions may help to improve understanding of the information provided, provide an opportunity to clear up any misconceptions, and aid the provision of information tailored to the specific needs of the patient; all of which can help to obtain valid consent.

Only 4.2% of the study participants had received written information and less than a third (24.2%) signed consent forms. This finding concurs with available data regarding informed consent practices among dental practitioners at the National Referral Hospital in Uganda, where only 5.3 % of them obtained written consent [19]. Though the current code of ethics by the Uganda Medical and Dental Practitioners Council (UMDPC) lacks details regarding the applicability of the different types of consent and only states that consent may be verbal or consent, most international ethical guidelines state that informed consent for invasive procedures should be written [16]. In addition, written rather than verbal consent with clearly presented information is a legal document that certifies a patient is fully aware of the nature and benefits of treatment and its potential consequences, along with any risk of failure and the estimated treatment cost [6]. The provision of written information and having the patient sign the forms have several advantages including the provision of document information the patient can refer to with the potential of increasing understanding, offering an opportunity for continuous onset, and helping to solve the problem of a significant decline in information remembered over time and could be used in case of litigation [8].

Most of the participants in the present study were satisfied or very satisfied with the information received during the consent process despite only half of them having received adequate information. This is comparable to results from similar studies: 96.5% and 92.0% of respondents were satisfied with the information received in Nigeria, and India [13,17] respectively. Similarly, Patil et al. observed that 93.5% of the patients were satisfied with the provided information despite their poor understanding of the informed consent [18]. This unexpected finding may be because most patients lack knowledge about dental treatments, their benefits, and associated risks; thus, they may not be aware when some information is not provided. However, dentists have an ethical and legal obligation to disclose adequate information to patients and to obtain valid informed consent.

Study strengths

The study provided baseline data for information disclosure about the consent process for fixed prosthodontic treatment among patients attending selected hospitals in Uganda. Sharing the study findings via seminars and workshops will allow dental facilities and the dental association to highlight areas for improvement, such as standardizing consent procedures and enhancing communication protocols. Such engagements attempt to promote compliance with informed consent standards and improve oral healthcare services. The strengths of this study included the large sample size that was distributed across three hospitals.

Study limitations

Limitations of the study included the lack of documentation with regard to what was discussed during the informed consent process for all participants, as this study used patient recall as a reporting tool. However, the informed consent process was observed for five participants at each hospital in an attempt to triangulate the data. The data cannot be generalized to represent Uganda as the study participants were from few dental facilities, however, with this reservation, the findings may still apply to informed consent practices in dental practices in Kampala and facilities in similar urban areas.

## Conclusions

The present study reported on the inadequacy of information provided during the informed consent process for fixed prosthodontic treatment, especially regarding risks, complications, and alternative treatment. There is a need to improve the manner in which informed consent is obtained, particularly in relation to the provision of comprehensive information regarding all facets of pre-and post-treatment procedures. The introduction of a standardized informed consent document that includes all pertinent information is recommended, together with additional visual aids to enhance the delivery of information and to give the patients a chance to ask questions.

## Appendices

**Table 6:** Questionnaire for assessment of information provided and patient satisfaction during the consent process for fixed prosthodontic treatment in selected dental facilities in Kampala City

Section I		Socio-demographic data			
Date			Study No		
Facility			Age		
Type of facility			☐ Government	☐ PNFP	
Sex			☐ Male	☐ Female	
Marital status			☐ Single	☐ Married	
			☐ Widowed	☐ Separated/divorced	
Religion			☐ Catholic	☐ Protestant	
			☐ Protestant	☐ Muslim	
			☐ Other		
The highest level of formal education attained			☐ None	☐ Primary	
			☐ Secondary	☐ Tertiary	
Ability to read and write			☐ Yes	☐ No	
Occupation		☐ None	☐ Studying	☐ Business/Self-employed	
		☐ Farming	☐ Civil/public service /formal employment	☐ Unemployed	☐ Other
Fixed prosthodontic treatment			☐ Crown (Cap)	☐ Bridge (fixed new tooth)	
			☐ On-lay	☐ Inlay	
			☐ Other, please specify		
The person who provided consent information for the treatment			☐ Dentist who performed treatment	☐ Other dentists in the practice	
			☐ Receptionist	☐ Chair-side assistant or dental nurse	
			☐ Chair side assistant or dental nurse	☐ Do not know	
Timing of providing consent information			☐ Prior appointment	☐ Before starting the procedure or on the day of treatment	
			☐ While conducting treatment	☐ During several occasions/ More than one of the occasions above	
Language(s) used when providing the explanations			☐ English	☐ Local language	
			☐ Both		
How was the information given to you?			☐ Oral	☐ Written, e.g., brochure	
			☐ Dental models	☐ Pictures	
			☐ Videos	☐ Other, please specify	
History of previous FPT: Have you ever had any of the following dental treatments (crown, bridge, inlay, or on-lay)?			☐ Yes	☐ No	
Section II		This section is to determine the adequacy of the information given and patient satisfaction with the information received.			
What information was received					
1	Which procedure are you going to have?			☐ Crown(Cap)	☐ Bridge(new fixed tooth)
				☐ Veneer (Artificial build-up of your tooth)	☐ Inlay
				☐ On-lay	☐ Other
				☐ Don’t know	
2	Were you informed about the nature of your dental problem (diagnosis)?			☐ Yes	☐ No
3	Were you informed of why it is necessary to have this treatment?			☐ Yes	☐ No
4	Were you informed about the details of the planned procedure you are going to have?			☐ Yes	☐ No
5	Were you informed who would perform the procedure?			☐ Yes	☐ No
5	Were the potential benefits of this treatment explained?			☐ Yes	☐ No
6	Were potential risks and inconveniences of the procedure explained to you?			☐ Yes	☐ No
7	Were any post-operative issues such as complications explained?			☐ Yes	☐ No
8	Were you informed about any other alternative treatment options?			☐ Yes	☐ No
9	Were you informed about how much the treatment is going to cost?			☐ Yes	☐ No
10	Were you informed about how long it is going to take to complete the procedure and your treatment?			☐ Yes	☐ No
11	Were you given a chance to ask questions about this treatment?			☐ Yes	☐ No
12	Were you told that you had the right to refuse or defer the decision to have this treatment?			☐ Yes	☐ No
13	Were you given a consent form to sign?			☐ Yes	☐ No
14a	Were you satisfied with the amount of information you received?			☐ Yes	☐ No
b	How satisfied are you with the information you received before this treatment?			☐ Very satisfied	☐ Satisfied
				☐ Neutral	☐ Low
				☐ No opinion	☐ No opinion
15	Please mention any other specific information you would have liked to receive before this treatment.				
This ends our interview, thank you for your time and assistance.					
